# microRNA-206 is involved in survival of hypoxia preconditioned mesenchymal stem cells through targeting Pim-1 kinase

**DOI:** 10.1186/s13287-016-0318-z

**Published:** 2016-04-22

**Authors:** You Zhang, Wei Lei, Weiya Yan, Xizhe Li, Xiaolin Wang, Zhenao Zhao, Jie Hui, Zhenya Shen, Junjie Yang

**Affiliations:** Department of Cardiology of the First Affiliated Hospital, Soochow University, Suzhou, China; Institute for Cardiovascular Science & Department of Cardiovascular Surgery of The First Affiliated Hospital, Soochow University, Suzhou, China; Department of Cardiovascular Surgery, Affiliated Shanghai 1st People’s Hospital, Shanghai Jiaotong University, Shanghai, China; Department of Thoracic and Cardiovascular Surgery, Northern Jiangsu People’s Hospital, Yangzhou, China

**Keywords:** Hypoxia, Mesenchymal stem cells, Pim-1, miR-206, Apoptosis

## Abstract

**Background:**

Overexpression of Pim-1 in stem/progenitor cells stimulated cell cycling and enhanced cardiac regeneration in vivo. We proposed that hypoxic preconditioning could increase survival of bone marrow mesenchymal stem cells (MSCs) via upregulation of Pim-1 and aimed to determine the microRNAs that modulate the expression of Pim-1.

**Methods and results:**

MSCs were subjected to hypoxia exposure. The expression of Pim-1 in MSCs was enhanced in a time-dependent manner, detected by quantitative PCR and western blot. miR-206 is predicted as one of the potential microRNAs that target Pim-1. The expression of miR-206 was decreased in hypoxic MSCs and reversely correlated with Pim-1 expression. Luciferase activity assay further confirmed Pim-1 as a putative target of miR-206. In addition, gain and loss-of-function studies with miR-206 mimics and inhibitors showed that inhibition of miR-206 in hypoxic MSCs promoted the migration ability of the cells, prevented cell apoptosis, and protected membrane potential of mitochondria, while the benefits were all blocked by Pim-1 inhibitor. In an acute model of myocardial infarction, transplanted hypoxic MSCs showed a significantly improved survival as compared with hypoxic MSCs overexpressing miR-206.

**Conclusions:**

Hypoxic preconditioning could increase short-term survival of bone marrow MSCs via upregulation of Pim-1, and miR-206 was one of the critical regulators in this process.

## Background

Pim-1, a proto-oncogenic serine–threonine kinase, was originally discovered as the proviral integration site for Moloney murine leukemia virus [1]. Pim-1 plays a role in the proliferation and survival of hematopoietic cells [2]. However, the role of Pim-1 in cardiac development has been overlooked for a long time. In 2007, Dr Mark A Sussman reported that Pim-1 could regulate cardiomyocyte survival downstream of Akt using Pim-1-deficient mice [3]. Also, overexpression of Pim-1 inhibited cardiomyocyte apoptosis and protected mice from infarction injury, which revealed the potential cardioprotective role of Pim-1. Successively, Sussman’s group showed that overexpression of Pim-1 in cardiac progenitor cells enhanced cardiac regeneration [4] and stimulated cell cycling [5]. Rejuvenation of stem cells by Pim-1 overexpression thus endued the cells with increased proliferative and regenerative potentials [6–8]. However, genetic modification of stem cells is not the first promising option in their utilization for tissue regeneration. In 2014, Hu et al. [9] reported that hypoxic preconditioning increases survival of cardiac progenitor cells via upregulation of Pim-1, discovering a method for nongenetic modification of Pim-1 in stem cells. In our study, we proposed that hypoxic preconditioning could increase survival of bone marrow mesenchymal stem cells (MSCs) via upregulation of Pim-1 and aimed to determine some microRNAs (miRNAs) that modulate the expression of Pim-1.

We demonstrated that hypoxia preconditioning enhanced the expression of Pim-1 kinase in MSCs in a time-dependent manner, as detected by quantitative PCR (qPCR) and western blot. We further predicted that microRNA-206 (miR-206) is one of the potential miRNAs that targets Pim-1 through TargetScan, microRNA, and miRDB software. In addition, miR-206 is a potential regulator of proliferation, apoptosis, and differentiation of pulmonary artery smooth muscle cells [10].

We confirmed that the expression of miR-206 was decreased in hypoxic MSCs. Next, qPCR analysis revealed that miR-206 reversely regulated the expression of Pim-1. Luciferase activity assay further confirmed Pim-1 as a putative target of miR-206. In addition, gain and loss-of-function studies with miR-206 mimics and inhibitors showed that inhibition of miR-206 in HP-MSCs promoted the migration ability of the cells, prevented cell apoptosis, and protected membrane potential of mitochondria, while the benefits were all blocked by Pim-1 inhibitor. In an acute model of myocardial infarction, transplanted HP-MSCs showed significantly improved survival as compared with hypoxic MSCs overexpressing miR-206. In conclusion, hypoxic preconditioning could increase short-term survival of bone marrow MSCs via upregulation of Pim-1, and miR-206 was one of the critical regulators in this process.

## Methods

### Animals

All animal procedures were approved by the Ethic Committee of Soochow University, Suzhou, China and were carried out in accordance with the Guidelines for the Care and Use of Research Animals established by Soochow University. Rats were housed at the Animal Facility of Soochow University on a 12-h light/dark cycle with free access to water and standard mouse food.

### Isolation and culture of bone marrow MSCs

Bone marrow MSCs were isolated from 4-week-old Sprague–Dawley rats. Briefly, the bone marrow was flushed from the femora of rats with low-glucose DMEM (Gibco, Gaithersburg, MD, USA), 10 % FBS (Gibco), and 1 % penicillin–streptomycin (HyClone, Logan, Utah, USA). Cells were collected and seeded onto a 10-cm diameter plate, and incubated in 5 % CO_2_ at 37 °C. After 24 h of incubation, the medium was changed first and replaced every 3 days thereafter. When at 80–90 % confluence, MSCs were dissociated with 0.25 % trypsin (Sangon biotech, Shanghai, China) and expanded at a 1:2 dilution. MSCs at passage 4 were used in all experiments.

### Hypoxia preconditioning of MSCs in vitro

For hypoxic culture, cells were cultured in a tri-gas incubator (Thermo Fisher Scientific, Marietta, OH, USA cell, Germany) composed of 94 % N_2_, 5 % CO_2_, and 1 % O_2_. To determine the optimal time length for hypoxic treatment, cells were cultured for 6, 12, and 24 h in hypoxia. MSCs were thus divided into four groups: normoxia, hypoxia for 6 h, hypoxia for 12 h, and hypoxia for 24 h. During the hypoxic preconditioning, MSCs were incubated with Quercetagetin (Calbiochem, San Diego, CA, USA), a specific Pim-1 activity inhibitor, at 10 μmol/l.

### Western blot

MSCs were lysed with lysis buffer including 1 % phenylmethane sulfonylfluoride (PMSF) and 1 % phosphatase inhibitors and the protein was extracted. The protein concentration was determined using a BCA Protein Assay Kit (Beyotime Biotechnology, Shanghai, China). The protein sample extracted from MSCs was separated by 10 % SDS-PAGE (for Pim-1 and β-actin) and transferred to a 0.45 mm nitrocellulose membrane (Millipore, Billerica, MA, USA) using a Trans-Blot SD Semi-Dry Electrophoretic Transfer Cell (Bio-Rad, Hercules, California, USA). Milk 5 % (w/v) in 0.1 % Tween 20/PBS was used to block the membranes for 1 h. The membranes were then incubated with rabbit polyclonal antibody against Pim-1 (1:100; Santa Cruz, Dallas, Texas, USA) and β-actin (1:1000; Beyotime Biotechnology) overnight and blotted with a HRP-linked secondary antibody (1:1000; Beyotime Biotechnology) for 2 h. Afterwards, the protein band could be visualized via enhanced chemiluminescence and analyzed by the Scion Image Software (Scion, Frederich, MD, USA).

### Target gene prediction

Three target prediction algorithms—TargetScan 6.2 (http://www.targetscan.org/), mirWALK (http://zmf.umm.uni-heidelberg.de/apps/zmf/mirwalk2/), and mircroRNA.org (http://www.microrna.org/microrna/)—were used to predict the potential target relationship between miR-206 and Pim-1.

### microRNA expression level in MSCs

Total RNA was extracted from rat MSCs using the PureLink™ RNA Mini Kit (Ambion, Foster City, CA, USA) and quantified using an ND2000 spectrophotometer (NanoDrop Technologies, Wilmington, DE, USA). The extracted RNA was subjected to RT-PCR using the Hairpin-it™ miRNAs RT-PCR Quantitation Kit (GenePharma, Shanghai, China). miRNA expression levels were measured using Step OnePlus Real-Time PCR System (Applied Biosystems, Foster City, CA, USA), according to the manufacturer’s instructions. miRNA expression was normalized to U6 snRNA.

### Dual-luciferase reporter assays

Using standard procedures, wild-type (WT) or mutant 3′-untranslated regions (UTRs) of Pim-1 were subcloned into the pmiR-RB-REPORT™ vector (Ribobio, Guangzhou, China) downstream of the luciferase gene. Pim-1-3′-UTR-WT or Pim-1-3′-UTR-Mut vectors were cotransfected with miR-206 mimics or negative control into MSCs, using Lipofectamine 2000 for 48 h. Firefly luciferase activity was measured at 48 h post transfection, using a Dual-Luciferase Reporter Assay System (Promega Corporation, Fitchburg, WI, USA), according to the manufacturer’s instructions. Each reporter plasmid was transfected at least three times, and each sample was assayed in triplicate.

### Transfection and groups in vitro

The miR-206 oligonucleotides including mimics, inhibitors, and negative control were purchased from GenePharma Co., Ltd (Shanghai, China). We dissolved the oligonucleotides with diethylpyrocarbonate (DEPC)-treated water to 50 nM. Cell transfection was performed with Lipofectamine 2000 (Invitrogen, Carlsbad, CA, USA), according to the manufacturer’s protocols. The efficiency of transfection was confirmed using RT-qPCR. Cells were then subjected to hypoxic treatment as already described. To provide a positive control on the blocking of Pim-1 activity, Quercetagetin (Calbiochem) was also added at 10 μmol/l when cells were under hypoxic exposure. The experimental groups are thus illustrated as follows: MSCs, HP-MSCs, HP-MSCs^miR-206^, HP-MSCs^anti-miR-206^, and HP-MSCs^anti-miR-206 + Pim-1 inhibitor^ (see Table 1).

**Table 1:** Groups used for the in-vitro and in-vivo experiments

Group^a^	Step 1	Time (h)	Step 2	Time (h)
MSCs	Untreated			
HP-MSCs	Untreated		Hypoxia exposure	12
HP-MSCs^miR-206^	Transfected with miR-206 mimics	48	Hypoxia exposure	12
HP-MSCs^anti-miR-206^	Transfected with miR-206 inhibitor	48	Hypoxia exposure	12
HP-MSCs^anti-miR-206 + Pim-1 inhibitor^	Transfected with miR-206 inhibitor	48	Hypoxia with Pim-1 inhibitor treatment	12

^a^MSCs subjected to different combinations of treatment

*Step 1* first step of treatment, *time* length of treatment, *Step 2* second step of treatment, *MSC* mesenchymal stem cell, *HP-MSC* mesenchymal stem cell subjected to hypoxic preconditioning, *miR-206* microRNA-206

### Transwell migration assay

Cell migration assays were performed using transwell filters with 8-μm pores (Fisher Scientific, Pittsburgh, PA, USA). Briefly, cells (5 × 10^4^ cells/200 μl) suspended in serum-free DMEM were plated into the upper compartment of a Transwell chamber in triplicate. Lower chambers were filled with 500 μl of DMEM containing 10 % FBS. After 4 h, cells were fixed in 4 % methanal for 20 min, stained with DAPI (Invitrogen) and counted under a fluorescent microscope (Olympus, Tokyo, Japan).

### Apoptosis assay

An Annexin V-fluorescein isothiocyanate (FITC) apoptosis detection kit (BD Pharmingen, San Diego, CA, USA) was used to perform the apoptosis assay. Briefly, 1 × 10^6^ cells were collected by trypsinization and resuspended in binding buffer containing Annexin V-FITC and propidium iodide. After incubation in the dark for 15 min, MSCs were analyzed using a BD FACS Aria flow cytometer.

### Measurement of the mitochondrial membrane potential

The mitochondrial membrane potential (∆*ψ*m) was measured using the lipophilic cationic probe JC-1 dye (Beyotime Biotechnology), according to the manufacturer’s instructions. Briefly, MSCs were stained with JC-1 (5 mol/l) at 37 °C for 20 min in the dark and rinsed three times with ice-cold working solution. The fluorescence was then monitored by the inversion fluorescence microscope (Olympus). The red fluorescence is caused by a potentially dependent aggregation in the mitochondria, reflecting ∆*ψ*m. Green emission of the dye represents the monomeric form of JC-1. Mitochondrial depolarization is indicated by a decrease in the red/green fluorescence intensity ratio.

### Acute myocardial infarction model of rat hearts and cell transplantation

Acute myocardial infarction (AMI) was made according to the method described previously [11]. Thirty female young (6–8 weeks) Sprague–Dawley rats (200–250 g) were studied. They were anesthetized once with by Avertin™ (300 mg/kg; Sigma, St. Louis, MO, USA) intraperitoneally. After a left lateral thoracotomy, the proximal portion of the left anterior descending artery was ligated with a 6–0 Prolene suture between the pulmonary artery outflow tract and the left atrium. Then, 1 million MSCs from male rats prepared in 70 μl suspension were transplanted by myocardial injection at multiple sites (3–4 sites/heart) in the free wall of the left ventricle during the acute phase of AMI. After injection, we closed the chests of the animals and the animals were allowed to recover. Four days later, the animals were euthanized for collection of the heart tissue samples for molecular studies.

### Detection of cell survival in vivo

Cell survival was determined by detection of *Sry* gene using qPCR in the myocardial tissue samples. Tissue samples were snap-frozen in liquid nitrogen and powdered. DNA purification was performed using the Genomic DNA Isolation kit (Qiagen, Germantown, MD, USA), and the concentration of the purified DNA was determined by spectrophotometry. The primer sequences for *sry* gene and β-actin were as follows: *sry* gene, forward 5′-GAGGCACAAGTTGGCTCAACA-3′ and reverse 5′-CTCCTGCAAAAAGGGCCTTT-3′; β-actin, forward 5′-CCACCATGTACCCAGGCATT-3′ and reverse 5′-ACTCCTGCTTGCTGATCCAC-3′.

### Statistical analysis

All data are shown as the mean ± standard error (SE). Differences between two mean values were evaluated by an unpaired Student two-tailed *t* test, and between three or more groups were analyzed using one-way analysis of variance by GraphPad Prism software (GraphPad Software Inc., San Diego, CA, USA). *P* < 0.05 was considered significant.

## Results

### Pim-1 expression was significantly increased in HP-MSCs

**Fig. 1:**
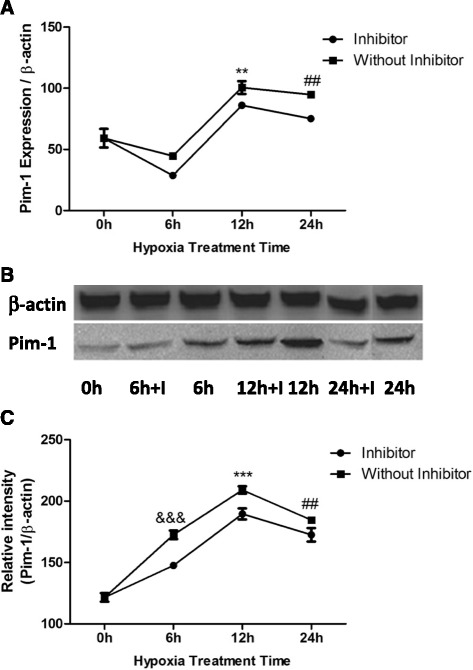
Hypoxia preconditioning changes the expression of Pim-1 time-dependently. **a** Expression of Pim-1 in MSCs at 0, 6, 12, and 24 h of hypoxia with or without Pim-1 inhibitor detected by qPCR (*n* = 4). **P < 0.01 vs 0 h and 6 h; ^##^ *P* < 0.01 vs 0 h and 6 h. **b** Western blot analysis of Pim-1 in MSCs with varied pretreatment. **c** Statistical analysis of Pim-1 intensity (*n* = 6). ^&&&^ *P* < 0.001 vs 0 h; ***P < 0.01 vs 0 h and 6 h; ^##^ *P* < 0.01 vs 0 h and 12 h

We first performed qPCR to detect the effect of hypoxic preconditioning on the expression of Pim-1 in MSCs. A time-course study was conducted at 0, 6, 12, and 24 h to quantify the Pim-1 expression in MSCs under hypoxic conditions (Fig. 1a). We observed a significant change of Pim-1 expression under low-oxygen conditions in a time-dependent manner. Although mildly decreased at 6 h, the level of Pim-1 expression in hypoxic MSCs was increased to about twofold at 12 h (*P* < 0.01 vs 0 h and 6 h), starting to decrease afterwards. It is worthy of note that the expression of Pim-1 was slightly inhibited by Quercetagetin, a specific inhibitor of Pim-1 activity (Fig. 1a). Furthermore, western blot analysis confirmed the tendency of Pim-1 expression (Fig. 1b, c). In contrast with its mRNA expression, the protein expression of Pim-1 significantly increased at 6 h under hypoxic conditions, suggesting a posttranscriptional regulation of the induction of Pim-1.

### Hypoxia pretreatment suppressed miR-206 expression and Pim-1 is a putative target of miR-206

**Fig. 2:**
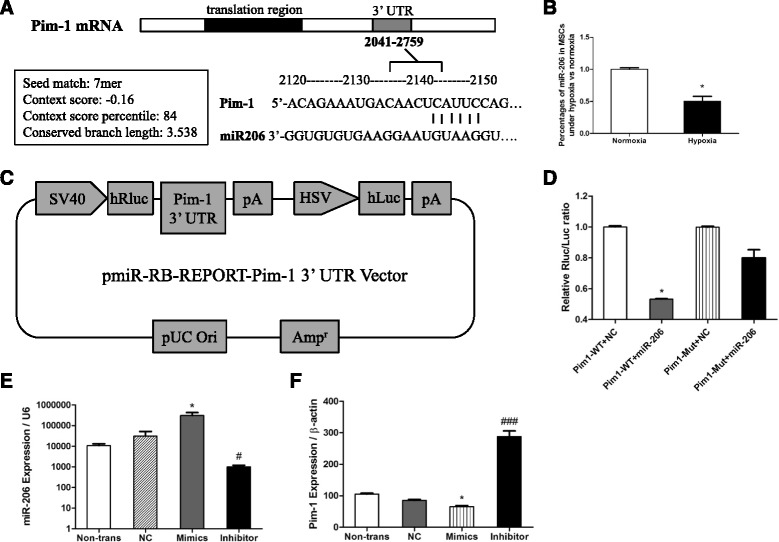
Pim-1 is a putative target of miR-206 in MSCs. **a** Binding motif (2136–2142) of miR-206 on Pim-1 3′-UTR (2041–2759). **b** Percentages of miR-206 expression in MSCs under hypoxia vs normoxia (*n* = 3). *P < 0.05 vs normoxia. **c** Construction of luciferase construct. **d** Relative Rluc/Luc ratio (*n* = 3). *P < 0.05 vs Pim-1-WT + NC. **e** Expression of miR-206 in MSCs after transfection of mimics and inhibitors (*n* = 6). *P < 0.05 vs non-trans, NC, and inhibitor; ^#^ *P* < 0.05 vs non-trans and NC. **f** Expression of Pim-1 in MSCs after transfection of miR-206 mimics and inhibitors (*n* = 6). *P < 0.05 vs non-trans; ^###^ *P* < 0.001 vs non-trans, *NC* negative control and mimics. *miR-206* microRNA-206, *MSC* mesenchymal stem cell, *UTR* untranslated region, *WT* wild type

miRNAs play a critical role in the posttranscriptional regulation of gene expression, and thus we could determine the miRNAs modulating Pim-1. Three databases (TargetScan, microRNA, and miRDB) were used to predict the putative miRNAs that target Pim-1, and miR-206 was predicted as one of the potential miRNAs binding on the 3′-UTR of the Pim-1 gene (Fig. 2a). To further confirm the targeting relationship between miR-206 and Pim-1, we first analyzed miR-206 expression using qPCR in HP-MSCs and found it was decreased to 50 ± 13 % under hypoxia vs normoxia (Fig. 2b). Furthermore, we constructed the luciferase reporter vector (Fig. 2c) to perform the luciferase reporter assay. As shown in Fig. 2d, miR-206 significantly decreased the relative luciferase reporter activity of the wild-type Pim-1 3′-UTR, whereas that of the mutant Pim-1 3′-UTR did not change significantly, which suggests that miR-206 could directly bind to the 3′-UTR of Pim-1. We then transfected MSCs with miR-206 mimics or miR-206 inhibitors, and over 90 % of the cells were successfully transfected. The expression of miR-206 was significantly upregulated or downregulated after transfection with mimics and inhibitors, respectively (Fig. 2e). qPCR showed that the inhibition of miR-206 in MSCs was concurrent with the increased expression of Pim-1 and vice versa (Fig. 2f). These results provided clear evidence that hypoxia pretreatment suppressed the expression of miR-206 and Pim-1 is a putative target of miR-206.

### Abrogation of miR-206 in HP-MSCs promotes the migration ability of the cells

**Fig. 3:**
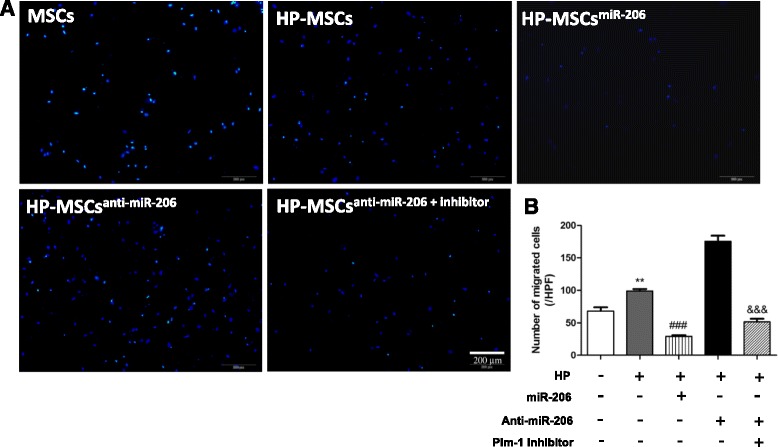
Hypoxia exposure of MSCs promotes the migration ability of the cells by modulation of miR-206/Pim-1. **a** Representative images of migrated cells stained with DAPI. *Bar*, 200 μm. **b** Numbers of migrated cells per high-power field (*HPF*) were statistically analyzed (*n* = 4). **P < 0.01 vs MSCs; ^###^ *P* < 0.001 vs HP-MSCs; ^&&&^ *P* < 0.001 vs HP-MSCs^anti-miR-206^. *miR-206* microRNA-206, *MSC* mesenchymal stem cell, *HP-MSC* MSC subjected to hypoxic preconditioning

The transwell assay was conducted to examine the migration abilities of MSCs under different conditions. MSCs without any pretreatment were used as a baseline control for all other experimental groups. As shown in Fig. 3a, the migration ability of HP-MSCs had a significant increase (*P* < 0.01 vs MSCs), but was inhibited when overexpressing miR-206 in HP-MSCs (*P* < 0.001 vs HP-MSCs). Besides, HP-MSCs with the inhibition of miR-206 had much more migrated cells, but when the cells were treated with Quercetagetin at the same time, migrated cells were reduced by about 70 % (*P* < 0.001 vs HP-MSCs^anti-miR-206^) (Fig. 3b). This suggests that hypoxic preconditioning could promote the migration ability of MSCs by inhibiting miR-206 expression, and Pim-1 is a downstream target of miR-206.

### Cytoprotective effects of Pim-1/miR-206 in HP-MSCs

**Fig. 4:**
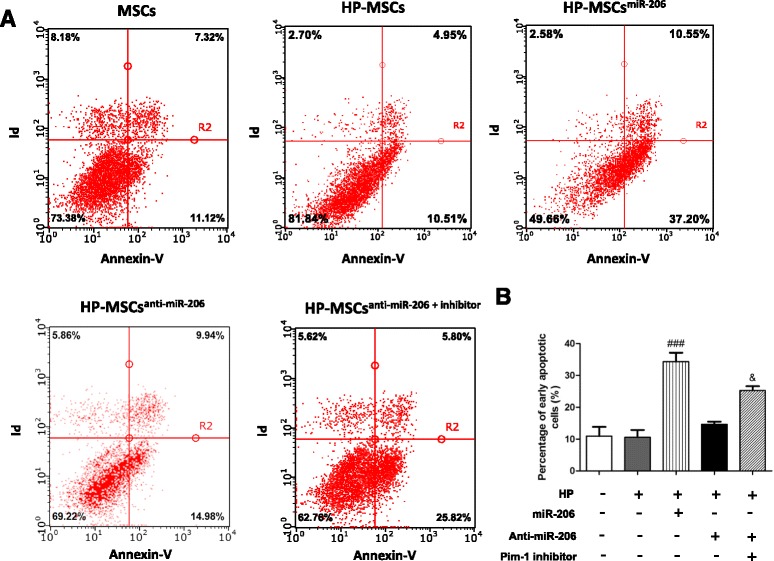
Anti-apoptosis effect of miR-206/Pim-1 in HP-MSCs. **a** Flow cytometry analysis of apoptotic cells in different groups. **b** Percentages of early apoptotic cells were statistically analyzed (*n* = 3). ^###^ *P* < 0.001 vs HP-MSCs, ^&^ *P* < 0.05 vs HP-MSCs^anti-miR-206^. *miR-206* microRNA-206, *MSC* mesenchymal stem cell, *HP-MSC* MSC subjected to hypoxic preconditioning

To further study the role of miR-206 and Pim-1 in cytoprotection of HP-MSCs, we examined the apoptosis of MSCs in different groups by flow cytometry analysis (Fig. 4a). Hypoxic pretreatment slightly delayed the early apoptosis of the cells (*P* > 0.05 vs MSCs). However, HP-MSCs overexpressing miR-206 showed a higher apoptosis rate (*P* < 0.001 vs HP-MSCs) (Fig. 4b). In addition, Pim-1 inhibition in HP-MSCs after anti-miR-206 transfection significantly accelerated the early apoptosis of the cells (*P* < 0.05 vs HP-MSCs^anti-miR-206^), which further confirmed the importance of miR-206 and Pim-1 in cytoprotection of HP-MSCs and the biological relationship between them.

### Inhibition of miR-206 in HP-MSCs protects the membrane potential of mitochondria

**Fig. 5:**
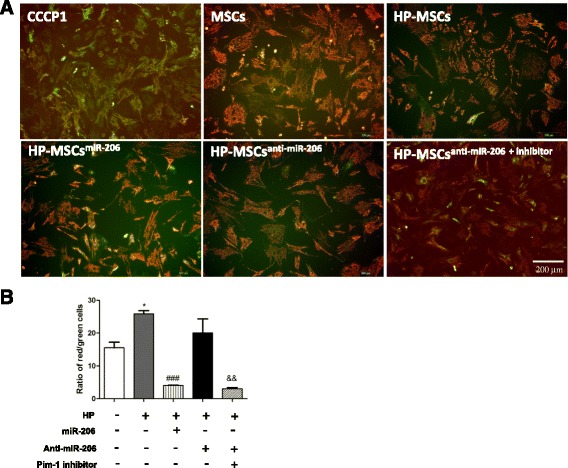
Inhibition of miR-206 in HP-MSCs protects membrane potential of mitochondria. **a** Representative images of mitochondria membrane potential stained by JC-1. Cells treated with CCCP-1 served as a control. *Bar*, 200 μm. **b** Ratios of red/green cells were calculated and analyzed (*n* = 4). *P < 0.05 vs MSCs; ^###^ *P* < 0.01 vs HP-MSCs; ^&&^ *P* < 0.001 vs HP-MSCs^anti-miR-206^. *miR-206* microRNA-206, *MSC* mesenchymal stem cell, *HP-MSC* MSC subjected to hypoxic preconditioning

The protective effect of Pim-1 in apoptosis via the mitochondrial pathway has been widely studied [12]. Therefore, we performed JC-1 staining to examine the protective effects of miR-206 and Pim-1 on mitochondrial integrity. The red fluorescence of JC-1 is caused by a potentially dependent aggregation in the mitochondria, reflecting ∆*ψ*m. Green emission of the dye represents the monomeric form of JC-1. Mitochondrial depolarization is indicated by a decrease in the red/green fluorescence intensity ratio. As shown in Fig. 5a and 5b, hypoxia exposure of MSCs significantly decreased mitochondria depolarization compared with untreated MSCs (*P* < 0.05 vs MSCs). However, the red/green ratio of JC-1 was remarkably decreased when HP-MSCs were transfected with miR-206 mimics (*P* < 0.01 vs HP-MSCs). In addition, Pim-1 inhibition in HP-MSCs after anti-miR-206 transfection decreased the red/green ratio of JC-1 from 20 ± 7 % (HP-MSCs^anti-miR-206^) to 3 ± 0.6 % (HP-MSCs^anti-miR-206 + inhibitor^) (*P* < 0.001).

### HP-MSCs with lower miR-206 showed improved survival in the infarcted heart

**Fig. 6:**
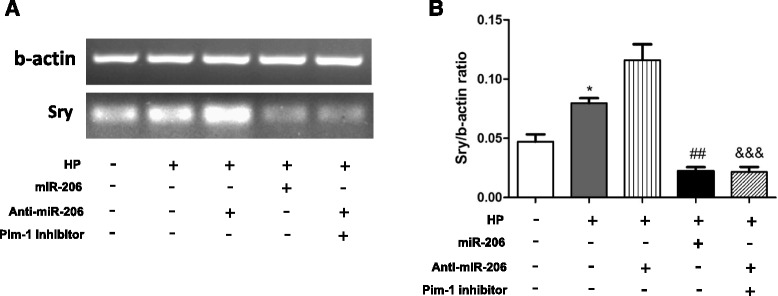
HP-MSCs with lower miR-206 showed improved survival in the infarcted heart. **a** Electrophoresis analysis of *sry* gene in the infarcted hearts 4 days post transplantation. **b** Ratios of *Sry*/β-actin were calculated and analyzed (*n* = 4). *P < 0.05 vs MSCs; ^##^ *P* < 0.01 vs HP-MSCs; ^&&&^ *P* < 0.001 vs HP-MSCs^anti-miR-206^. *HP* hypoxic preconditioning, *miR-206* microRNA-206

MSCs from male donors were transplanted to female rats subjected to myocardial infarction. The survival of transplanted cells was evaluated by detection of *Sry* gene in the ischemic hearts as described previously [13]. Hearts were collected 4 days after cell transplantation, and cell survival was examined (Fig. 6a). In vivo, MSCs survived more by hypoxic preconditioning (*P* < 0.05 vs MSCs), which was prevented by overexpression of miR-206 (*P* < 0.01 vs HP-MSCs) (Fig. 6b). As it was observed in vitro, inhibition of miR-206 in HP-MSCs showed improved survival, which was prevented by Pim-1 inhibitor (*P* < 0.001 vs HP-MSCs^anti-miR-206^).

## Discussion

The important findings of our study include the following: Pim-1 kinase is upregulated in MSCs under hypoxic conditions; miR-206 plays a mechanistic role in the migration and survival of MSCs via its putative target Pim-1; the prosurvival effect of miR-206/Pim-1 maintains the mitochondria membrane potential during hypoxic treatment of MSCs; and abrogation of miR-206 in hypoxic MSCs enhanced survival of the cells in the ischemic myocardium.

The role of Pim-1, a proto-oncogenic serine–threonine kinase, in cardiac development has been overlooked for a long time. Dr Sussman first discovered that Pim-1, downstream of Akt, regulates cardiomyocyte survival [3]. Regenerative therapies utilizing stem/progenitors cells engineered with Pim-1 enhanced regenerative potential of the cells [4, 8, 14], thus making Pim-1 an important player in the treatment of severe heart failure. Very recently, it was reported that hypoxic preconditioning increases survival of cardiac progenitor cells via upregulation of Pim-1, discovering a method for nongenetic modification of Pim-1 in stem cells. Another report [15] also claimed that Pim-1 could promote MSC proliferation and prevent MSC apoptosis. Thus we raised a proposal that hypoxic preconditioning could increase survival of MSCs via upregulation of Pim-1. Not surprisingly, we demonstrated that Pim-1 was gradually increased, reaching a peak at 12 h of hypoxia. Next, we aimed to determine the miRNAs that modulate the expression of Pim-1 in MSCs under hypoxic conditions. Through the three target gene prediction software packages, we predicted that miR-206, miR-328, miR-327, miR-532-3p, and miR-760-3p had putative binding sites on the 3′-UTR region of Pim-1. Through literature searching, we screened out miR-206 and miR-328 since they were reported to be downregulated in hypoxic situations [16, 17]. miR-206 was further confirmed to be downregulated in hypoxic MSCs, while miR-328 was not changed (data not shown). Next, the targeting relationship of miR-206 and Pim-1 was validated by qPCR and luciferase reporter activity.

Although little is known about miR-206 for its function during the preconditioning of stem cells, miR-206 has been mostly studied for its association with the pathogenesis of human cancers. A number of studies have shown that miR-206 is frequently downregulated in many human malignancies, including colorectal cancer [18], cervical cancer [19], lung cancer [20], gastric cancer [21], and breast cancer [22], and is associated with a malignant phenotype. These studies have validated that upregulation of miR-206 inhibited cancer cell proliferation and migration, blocked the cell cycle, and activated apoptosis. In our study, we observed that during the hypoxic preconditioning of MSCs, the inhibition of miR-206 had anti-apoptotic and migration-promoting effects on the MSCs, which were blocked either by overexpression of miR-206 or by abrogation of Pim-1 activity using the specific inhibitor, consistent with the observations in cancer research. In HeLa and C2C12 cells, miR-206 targets NOTCH3 expression to induce cell-cycle arrest and the inhibition of tumor cell migration [23]. It was also shown that through the c-Met pathway, miR-206 effectively inhibited gastric cancer progression [21]. In this study we provide the first evidence that hypoxic preconditioning regulated the expression of miR-206, which plays an essential role in mediating migration and apoptosis by targeting Pim-1 kinase in MSCs.

A loss of mitochondrial membrane potential is the initial manifestation of mitochondrial damage. In the apoptosis pathway, mitochondrial membrane potential was impaired first, and then apoptosis cascade starts [24, 25]. In this study, the protective effect of Pim-1/miR-206 in HP-MSCs maintains the membrane potential of mitochondria, slowing down the progression of apoptosis. In addition, hypoxic treatment significantly enhanced survival of MSCs after transplantation in ischemic hearts, while the pretreatment of MSCs with miR-206 and Pim-1 inhibitor significantly abolished the cytoprotective effects of preconditioning.

## Conclusions

We demonstrate that miR-206 is modulated by hypoxic preconditioning of MSCs through targeting Pim-1 kinase, elucidating a new aspect of prosurvival signaling in hypoxia. Moreover, the protective effects of miR-206/Pim-1 in HP-MSCs supported their short-term survival under ischemic stress after transplantation. miR-206/Pim-1 may serve as a molecular therapeutic strategy in stem cell translational research.

### Availability of data and materials

The datasets supporting the conclusions of this article are included within the article.

## Abbreviations

AMI: acute myocardial infarction
∆*ψ*m: mitochondrial membrane potential
HP-MSC: mesenchymal stem cell subjected to hypoxic preconditioning
miR-206: microRNA-206
miRNA: microRNA
MSC: mesenchymal stem cell
qPCR: quantitative PCR
UTR: untranslated region
WT: wild type
